# Nursing and Midwifery Students' Satisfaction with Their Clinical Rotation Experience: The Role of the Clinical Learning Environment

**DOI:** 10.1155/2021/7258485

**Published:** 2021-04-14

**Authors:** Alhassan Basour Adam, Andrew Adjei Druye, Akwasi Kumi-Kyereme, Wahab Osman, Afizu Alhassan

**Affiliations:** ^1^Department of General Nursing, School of Nursing and Midwifery, University for Development Studies, Tamale, Ghana; ^2^School of Nursing and Midwifery, University of Cape Coast, Cape Coast, Ghana; ^3^Department of Population and Health, University of Cape Coast, Cape Coast, Ghana; ^4^Department of Advance Nursing, School of Nursing and Midwifery, University for Development Studies, Tamale, Ghana; ^5^Ghana College of Nurses and Midwives (GCNM), 214 Residential Area, West Legon, Accra, Ghana; ^6^Kpembe Nursing and Midwifery Training College, Kpembe, Ghana

## Abstract

**Background:**

The clinical learning environment and clinical rotation experience of students are integral to nursing curriculum and are a crucial component of nursing education which helps transform theoretical knowledge to clinical practical skills.

**Objective:**

This study was aimed at assessing the role of the clinical learning environment on undergraduate nursing and midwifery students' satisfaction with their clinical rotation experience.

**Method:**

The study employed a quantitative cross-sectional survey design. Data was collected from a sample of 240 undergraduate nursing and midwifery students of the University for Development Studies, Tamale, Ghana, using a structured questionnaire. Ethical approval was obtained from the University of Cape Coast Ethics Review Board. Descriptive analysis was displayed as frequencies and percentages. Inferentially, Fisher's exact test, linear regression, and Spearman's correlation tests were used to test for and quantify associations between independent and dependent variables at *p* ≤ 0.05.

**Results:**

The level of students' satisfaction with both clinical rotation experience and the clinical learning environment was high (65.6% and 63.5%, respectively). A statistically significant association of the students' satisfaction with their clinical rotation experience was found. There was a statistically significant relationship between the clinical learning environment (*χ*^2^ (9, *N* = 224) = 80.665, *p* < 0.001), pedagogical atmosphere in the clinical area (*r*_*s*_ = 0.379, *p* < 0.001), the leadership style of the ward manager (*r*_*s*_ = 0.340, *p* < 0.001), the premises of nursing in the ward environment (*r*_*s*_ = 0.501, *p* < 0.001), and the students' satisfaction with their clinical rotation experience.

**Conclusion:**

These findings provide nurse educators and clinicians with meaningful understanding about areas to prioritise when planning clinical learning opportunities in such a way that skills learning and practice of nursing skills are successful and satisfactory for undergraduate student nurses and midwives.

## 1. Background

Clinical rotation experience is crucial in nursing and midwifery education as it helps student nurses and midwives to transform theoretical knowledge into clinical practice. Clinical rotation that exposes students to a supportive clinical learning environment (CLE) influences nursing and midwifery students' satisfaction, knowledge, skills, attitudes, and interest in their training [1–4].

Clinical rotation experience is a planned experience for a specific nursing educational course, and experiences gained by the students in hospitals, clinics, and health care centers and in the community [5, 6]. Clinical rotation is an important exercise because it offers students the opportunity to combine cognitive, psychomotor, and affective skills that influence students' satisfaction, knowledge, skills, attitudes, and interest in nursing patients [5, 7].

Clinical rotation experience is achieved in a clinical learning environment, which consists of all the clinical environs of the nursing and midwifery students such as the clinical settings, the staff nurses and midwives, the patients, the nurse preceptors, and the equipment. Exploration of such environments provides insight into the educational functioning of the students in clinical areas and allows nursing tutors to provide opportunities for students learning in the clinical setting [8, 9]. Clinical learning environment is characterized by pedagogical atmosphere of the clinical environment, leadership style of the ward managers, premises of nursing, and the supervisory relationships between students nurses, clinical staff, and nurse educators [10].

The pedagogical atmosphere of the clinical environment can be positive or negative. A positive pedagogical environment is characterized by nonhierarchical structure, teamwork, good spirits, and interpersonal communications [10]. The positive pedagogical environment allows students to be motivated, feel involved in ward activities, have good relationships with other team members, and explore practices [7, 11].

The leadership style of the ward manager remains a crucial element of experiential learning in the clinical setting [12]. A good learning environment is characterized by a democratic leadership style, where the ward manager is aware of the physical and emotional needs of the nursing staff and students and stimulates participation in a wide range of experiences that promote learning [4, 13].

The premises of nursing, where nursing care and learning occur, consist of the culture and values of nursing in the ward, information flow related to patient's care, documentation of nursing care plans, recording of nursing procedures, and sufficient meaningful learning situations on the ward [14]. Cultural and organizational factors in the ward such as ethical principles, hospital etiquettes, empathy, caring, teamwork, and socialization within the profession often influence students' rotation experience and foster skills acquisition and independent critical thinking [15].

The most effective premises of nursing environment for clinical learning is one that is supportive, free from fear, and encourages openness and respect for the student as an individual [16]. Good premises also recognize students as younger colleagues rather than strangers and focus on student learning needs rather than only health care service [17, 18]. In such an environment, students can develop self-confidence, competence, good interpersonal communication, and problem-solving skills, which can enhance their clinical rotation experience [19].

Despite the importance of clinical learning environment (health facilities) in contributing to trainees satisfaction with clinical rotation, it can be a major source of anxiety and stress among nursing students worldwide [9, 20, 21]. The clinical environment can be very difficult to control and predict due to a number of stimuli [18]. Additionally, health facilities primary concern is patient care rather than student learning. This can compromise students' learning and satisfaction with their clinical learning environment.

As a result of the multifaceted nature of the clinical learning environment, students have difficulty identifying potential learning opportunities, and consequently, some student feel overwhelmed [18, 22, 23]. Ineffective communication, inadequate readiness, and emotional reactions among others are challenges nursing and midwifery students face in the clinical learning environment [24].

In some clinical environments, nursing and midwifery students are mostly treated unfairly and, in extreme cases, discriminated against. For example, Jamshidi et al. [24] concluded that discrimination in the clinical environment is apparent in the behaviour of some nurses towards nursing and midwifery students, where medical students are given preferential treatment against nursing students [24]. Similarly, lack of adequate teaching and learning support for nursing and midwifery students, theory-practice gap, and poor interpersonal relationships between students and nursing staff in the ward have been reported by Mabuda, Potgieter, and Alberts [25]. These are barriers to constructive learning in the clinical learning environments. As a result of these, students usually are not satisfied with their clinical rotation, and their learning objectives set by the nursing faculty are usually not met [26].

While several research works have been published on undergraduate nursing and midwifery students' satisfaction with their experiential or clinical learning and the clinical learning environment in the developed countries such as Australia, Finland, Canada, and other places like Iran [1, 27–31], there are relatively few studies on this subject in the developing world.

In Ghana, nursing and midwifery education has been reported to be experiencing many challenges since time immemorial such as poor working relations between clinical environment and health training institutions, poor clinical environment, inadequate preceptor preparation, and inadequate students' supervision among others during clinical placements in health facilities [32, 33]. This could partly be blamed on inadequate assessment of the clinical environment and its dimensions in order to ascertain areas that support experiential or clinical learning, and those which require improvement. Providing an effective clinical learning environment to nursing and midwifery students ensures practical skills acquisition and promotes the students' satisfaction with the clinical rotation experience, which can result in production of highly trained and clinically competent nursing workforce.

Despite the acknowledged importance of clinical learning environment and clinical rotation on the acquisition of clinical nursing skills during training of nursing and midwifery students, research on student satisfaction with their clinical rotation and the clinical learning environment has not been adequately addressed in most countries in the developing world like Ghana.

### 1.1. Aim of the Study

This article, therefore, sought to provide answers to the degree to which nursing and midwifery students at the University for Development Students, Tamale, Ghana, are satisfied with their clinical rotation experience, as well as the clinical learning environment's role in their satisfaction with their clinical rotation experience.

### 1.2. Hypothesis

To achieve the aim of the study, the following null and alternate hypotheses were formulated:

H_0_: there is no statistically significant association between the clinical learning environment and students' level of satisfaction with their clinical rotation experience.

H_1_: there is a statistically significant association between the clinical learning environment and students' level of satisfaction with their clinical rotation experience.

## 2. Methodology

### 2.1. Study Design

The cross-sectional survey with a quantitative approach was used for this study. Both independent and dependent variables were measured at the same time as recommended by Bhattacherjee [34].

### 2.2. Study Setting

The study was conducted at the School of Nursing and Midwifery of the University for Development Studies, Tamale, Ghana. The University for Development Studies was established in 1992 as a multicampus institution as the fifth public University to be established in Ghana. The Tamale campus houses the School of Nursing and Midwifery, School of Medicine, and other allied health sciences. This setting was chosen because it is a tertiary institution, and the students were observed to have some challenges in the clinical environment during their clinical rotation practices in the clinical area.

### 2.3. Population and Sampling

The target population for the study were 715 undergraduate nursing and midwifery students in the third and fourth years of the School of Nursing and Midwifery of the University for Development Studies, Tamale, Ghana. These students have had exposure to different clinical settings and experiences and could provide valuable information by responding to the research questions. The sample size used for the study was 240, arrived at using Yamane [35] formula sample size formula *n*=*N*/(1+*N*(*e*)^2^) , where “*n*” is the sample size, “*N*” is the population size (715), and “*e*” is the margin of error or level of precision (0.05).

A proportional stratified random sampling technique was used to determine the number of students from each year group based on their total numbers. This approach was necessary to ensure equitable distribution of respondents with general nursing and midwifery background in the sample. The participants were divided into two categories, general nurses and midwives. Then a number of student nurses in each category was chosen based on the proportion of nursing and midwifery students from that category in the total population. Thus, general nurses who constituted 70% of the total student population were 157 students, whiles midwifery students were 67 representing 30% of the total student population.

### 2.4. Inclusion/Exclusion Criteria

Both postdiploma and generic students, who were regular students and had satisfactorily completed a minimum of two clinical rotation experiences, were included in the study. Students excluded were those in first and second year, as well as those on the distant learning program. This was because these students might not have had enough clinical experience to provide valuable information needed in the study. Further, students who were on field placement in communities at the time of data collection were excluded from the study because they were not available for selection.

### 2.5. Data Collection Instrument

A structured questionnaire was used to collect data from the respondents. The instrument comprised three sections capturing information about the participants' demographic characteristics, their level of satisfaction with clinical rotation experience, and the clinical learning environment. Section A was designed to elicit information on demographic information of participants such as the age, gender, marital status, religion, ethnicity, financial support, category of nursing students, and the level of entry to the programme.

Section B measured student's satisfaction with clinical rotation experience, using a 22-item scale adapted from the Clinical Learning Environment Inventory by Chan [11]. Section C measured student's satisfaction with the clinical learning environment, using a 20-item scale drawn from Clinical Learning Environment Inventory and Nurse Teacher Evaluation Scale by Johansson et al. [36]. As the main dependent variables, clinical rotation experience, and clinical learning environment were primarily measured at ordinal level on Likert scale of 1-strongly disagree, 2-disagree, 3-neutral, 4-agree, and 5-strongly agree. Analysis of the pretest data of the instrument showed a Cronbach's alpha of 0.86, indicating a high validity [37].

### 2.6. Data Collection Procedure

Data collection was conducted among third- and fourth-year nursing students who met the inclusion criteria at the University for Development Studies, Tamale. The questionnaire along with the cover letters to introduce the study purpose and the rights of the participants were distributed to the participants by the researchers and two data collection volunteers with data collection experience. The information sheet contained clarification of ethical issues concerning confidentiality and anonymity and provided contact information for participants to reach out to for clarifications. Participants were contacted during break periods. Participants who agreed to participate in the study signed the informed consent form and were asked to complete the questionnaire within one week, on their own. The researcher and the volunteer data collectors retrieved the completed questionnaires from the students after a week in April, 2018.

### 2.7. Data Management

Of the 240 participants samples and given questionnaires to complete, 224 completed the survey, giving a questionnaire return rate of 93.3%. Upon retrieving the completed questionnaires, it was kept in separately labelled envelopes, based on the programmes of study of the respondents (general nursing and midwifery).

The questionnaires were then cleaned, coded, and entered into SPSS for Windows Version 23 for statistical analysis. On completing the entries, the questionnaires were placed into their labelled envelopes and locked in a cabinet, only accessible to the researchers. The SPSS data was passworded and a backed-up copy of it was kept on an external drive, which was also password protected for data security reasons.

### 2.8. Data Analysis

Analysis of the data was carried out in SPSS for Windows Version 23. For purposes of analysis and easy communication of findings, an average score of the results from the Likert scale items (1-strongly disagree, 2-disagree, 3-neutral, 4-agree, and 5-strongly agree) measuring satisfaction with clinical rotation experience and the clinical learning environment in the questionnaire was calculated. The level of respondents' satisfaction with their clinical rotation experience and the clinical learning environment was determined by taking an average of their total satisfaction score to determine whether they scored very low (1 to 27), low (28 to 54), high (55 to 81), or very high (82 to 11). The results were then displayed in tables as frequencies and percentages.

Inferentially, Chi-square and Fisher's exact tests were used to determine the association between selected demographic variables (age, gender, student category, and level of entry) and respondents' satisfaction with the clinical rotation experience and clinical learning environment. Where statistically significant associations existed, variables were entered into a logistic regression model to determine the strength of association between satisfaction with clinical rotation experience and the respective demographic predictors at the 95% confidence interval. A *p*-value of less than or equal to 0.05 was deemed statistically significant.

Further, a Spearman's correlation test was conducted to predict the association between students' satisfaction with clinical rotation experience and dimensions of the clinical learning environment such as pedagogical atmosphere of the ward environment, leadership style of ward managers, and premises of nursing in the ward.

### 2.9. Ethical Considerations

This study was conducted at the in Tamale, Ghana, at the School of Nursing and Midwifery of the University for Development Studies. Ethical approval was obtained from the Institutional Review Board of University of Cape Coast (Reference number: 0990–4279), and permission for data collection was obtained from the School of Nursing and Midwifery, University for Development Studies. Access to the participants was gained through the heads of departments of nursing and midwifery.

Prior to the commencement of questionnaire distribution to sampled participants, all participants were informed about the objectives and purpose of the study. An informed consent was given to each respondent to read, seek clarifications, and consent. Issues of anonymity and confidentiality of information provided and participants were ensured as they were not required to provide their names. Information about the voluntary nature of the study and their right to withdraw at any stage without any consequence were also explained. No adverse event or effect was expected or anticipated from participating in this study.

## 3. Results

With a completed questionnaire return rate of 93.3% of the 240 respondents sampled, a sample size of 224 was finally realized and used for all statistical analyses under this section.

### 3.1. Demographic Characteristics of Respondents

Table 1 presents the demographic characteristics of participants including age, gender, marital status, religion, ethnicity, and financial support of the participants. Majority of the participants were in their 20s (mean age of 23.8 years). Female were more (58.5%) than males (41.5%). General nursing students were more (71.4%) than midwifery students (28.6%). Majority of students were generics (79%) and a small percentage were postdiploma nursing students (21%).

### 3.2. Respondents' Satisfaction with Their Clinical Rotation Experience

Statistics about the students' level of satisfaction with their clinical rotation experience is presented in Table 2. Most of the students (65.6%) rated their satisfaction with clinical rotation experience as high or very high, while 21% rated it as low or very low.

A Fisher's exact test was performed to determine the association between some selected demographic variables of respondents and their level of satisfaction with clinical rotation experience showing no statistically significant association as illustrated in Table 3.

### 3.3. Respondents' Satisfaction with the Clinical Learning Environment

Close to two-thirds (63.5%) of the respondents were in the category of those who said they had high levels of satisfaction with the clinical learning environment. Only a few (0.4%) of the respondents indicated that they had very low satisfaction with clinical learning environment. The details of these have been illustrated in Table 4.

### 3.4. Role of the Clinical Learning Environment in Respondents' Satisfaction with Their Clinical Rotation Experience

A Fisher's exact test showed that there was a statistically significant association between the clinical learning environment and respondents' satisfaction with their clinical rotation experience (*χ*^2^ (9, *n* = 224) = 80.665, *p* < 0.001). As a result, we fail to accept the null hypothesis, which stated that “there is no statistically significant association between clinical learning environment and students' level of satisfaction with their clinical rotation experience” and accept the alternate hypothesis, which stated that “there is a statistically significant association between clinical learning environment and students' level of satisfaction with their clinical rotation experience”. The result of this test is presented in Table 3.

Using a linear regression model, the strength of the association between the clinical learning environment and respondents' satisfaction with their clinical rotation experience was tested. The regression analysis results, presented in Table 5, show that *R*^*2*^ was 0.422, which means that the clinical learning environment accounts for 42.2% of the variance in respondents' satisfaction with their clinical rotation experience at *p* < 0.001. Additionally, the “B” value was 0.511, representing the amount of increment in respondents' satisfaction with their clinical rotation experience for every unit increase in respondents' satisfaction with the clinical learning environment.

Further analysis was conducted to quantify the relationship between the dimensions of clinical learning environment and respondents' satisfaction with clinical rotation experience using Spearman's correlation test (see Table 6). All three dimensions of the of clinical learning environment (pedagogical atmosphere of the ward, leadership style of ward manager, and premises of nursing in the ward environment) had statistically significant relationship with respondents satisfaction with their clinical rotation experience at *p* ≤ 0.05. Pedagogical atmosphere of the ward environment had a moderate positive correlation (*r*_*s*_ (224) = 0.379, *p* < 0.001), and leadership style of ward manager also had a moderate positive correlation (*r*_*s*_ (224) = 0.340, *p* < 0.001), while the premises of nursing in the ward environment had a strong positive correlation (*r*_*s*_ (224) = 0.501, *p* < 0.001) with respondents' satisfaction with their clinical rotation experience.

## 4. Discussion

### 4.1. Respondents' Satisfaction with Their Clinical Rotation Experience

The findings from this study showed that nursing students generally have high levels of satisfaction with their clinical rotation experience. Many of them fell in the category of high satisfaction group, whereas a small number was in the category of very low satisfaction. This finding is similar to that of Chuan and Barnett [29], who found that nursing and midwifery students reported high levels of satisfaction with their clinical rotation. A similar study by Al-Sebaee et al. [1] also reported that majority of nursing students expressed high levels of satisfaction with their clinical rotation experience, and only a minority were those who had low level of satisfaction.

Al-Sebaee et al. [1] explained that students' satisfaction with their clinical experience was mainly because they met their rotation objectives, enjoyed their time, and worked with a team who were willing and available to assist them in learning. Further, the students' needs were matched with their preceptors in the clinical area. This view is supported by Alspach [38], who indicated that an optimal satisfaction and orientation are best achieved when the needs of the nursing and midwifery students are matched with the competencies of the preceptors. Another study by Zilembo and Monterosso [39] also confirmed that learning from experienced, knowledgeable, and competent nurse preceptors exposes nursing and midwifery students to effective clinical experience, which directly enhances the students' satisfaction with the clinical rotation.

This study also found that when students were asked to indicate if the clinical rotation was a waste of their time, most indicated that it was not a waste of time. However, a small number indicated it was actually a waste of time. This finding is in line with findings by Perli and Brugnolli [40], who found in their study that nursing students overall rated their clinical rotation experience in the clinical learning environment high. Third-year students were extremely satisfied with activities done on the ward. All the students agreed that they were highly satisfied with the clinical rotation experience and deemed it as useful and not a waste of time.

### 4.2. Respondents' Satisfaction with the Clinical Learning Environment

Findings about nursing and midwifery students' satisfaction with the clinical learning environment show that close to two-thirds of the students were highly satisfied with the clinical learning environment. This finding is comparable to findings by Papastavrou et al. [4] in a study in Cyprus where nursing students were found to be highly satisfied with their clinical learning environment. The researchers attributed this finding to the level of motivation, the nursing care delivery, the supervisory relationship with the mentor, and nurse teachers' role in the clinical practice area.

The findings of this current study, which shows a high level of satisfaction by nursing students with their clinical learning area, are similar to findings by Nepal et al. [41] in a Nepalese study. The findings of this current study further confirm previous studies in Europe by Papastavrou et al. [4], Saarikoski et al. [42], and Saarikoski and Leino-Kilpi [10], despite the different nursing education systems and settings.

The high levels of satisfaction with the clinical learning environment by nursing students in this current study could be attributed to a number of reasons. This could be due to the presence of preceptors at Tamale Teaching Hospital, which is the main clinical learning environment for nursing and midwifery students of the University for Development Studies, more so, the clinical staff being well trained and ready to assist students, and the recognition of students as part of the health care team and being treated with utmost respect and appreciation. Another ground on which the students could demonstrate these high levels of satisfaction with the clinical learning environment has to do with effective levels of clinical nursing skills teacher guidance, constant feedback on student's clinical performance, and regular clinical conferences with clinicians and nurse teachers. Similarly, the degree of satisfaction also appeared to be influenced by the unique organizational atmosphere of the ward and hospital environment at Tamale Teaching Hospital, with well-structured clinical environment to support students' learning.

On the contrary, the findings of this study are at variance with an Iranian study by Hakim [43], who established that most nursing students had little satisfaction to the situations of their clinical learning environment. An earlier study in 2013 also reported low levels of students' satisfaction with their clinical learning environment [44].

Another finding from this study is that the clinical learning environment is found to have great influence on undergraduate nursing and midwifery students' satisfaction with their clinical rotation experience. Many studies have demonstrated the importance of the clinical learning environment in students' satisfaction with their clinical rotation. Perli and Brugnolli [40] as well as D'Souza et al. [45] all found that the clinical learning environment is considered an important influential factor for determining nursing students' satisfaction with their clinical rotation experience. Akta and Karabulut [46] further confirmed in a Turkish study that when nursing and midwifery students graduate without enough clinical rotation practice experience and with insufficient practical skills, then it may be attributed to poor and inadequate clinical learning environmental support to student learning. It is therefore obvious that student dissatisfaction with the clinical learning environment is one of the important factors that can hinder satisfactory clinical rotation experience of undergraduate nursing and midwifery student.

### 4.3. Association between Clinical Rotation Experience and Dimensions of Clinical Learning Environment

The findings from this current study generally show that students satisfaction with their clinical rotation experience was significantly related to all of the three dimensions of the clinical learning environment such as pedagogical atmosphere of the ward, leadership style of ward manager, and the premises of nursing in the ward environment. Our findings are similar to that of Papastavrou et al. [4] who found that nursing and midwifery students' satisfaction with clinical rotation experience was significantly associated to all of the three dimensions of the clinical learning environment.

One notable finding in this current study was that the premises of nursing in the ward environment had more influence on the nursing and midwifery students' satisfaction with their clinical rotation experience than pedagogical atmosphere and leadership style of managers. According to Skaalvik et al. [14], premises of nursing consist of the culture and values of nursing in the ward, information flow related to patients care, documentation of nursing care plans, recording of nursing procedures, and sufficient meaningful learning situations on the ward. In the nutshell, it is important that clinical nurses and midwives resist the temptations of shot-cuts to get the work done, since nursing and midwifery students may end up copying the wrong things.

However, this current finding differs from some findings of Papastavrou et al. [4] where they found that pedagogical atmosphere turns to have more influence on the students' satisfaction than the rest of clinical learning environment dimensions. The differences could be due to the differences in sample size as well as the study settings. Our sample size was lesser than that of Papastavrou et al.'s [30] study.

## 5. Conclusion

Based on the finding of this study, it can be concluded that the level of satisfaction of undergraduate nursing and midwifery students of the University for Development Studies, Tamale, Ghana, with their clinical learning environment was high. This could be due to the well-structured clinical learning environment, where the students obtain their clinical experience.

The clinical learning environment greatly influences nursing and midwifery students' satisfaction with their clinical rotation experience. The students indicated a high level of satisfaction with their clinical learning environment. This could be attributed to good institutional working relationship between University for Development Studies and Tamale Teaching Hospital, which is the major site where nursing and midwifery students obtain their clinical training.

In light of these findings from this study, it is clear that there are other factors influencing general satisfaction of undergraduate nursing and midwifery students' satisfaction with their clinical rotation experience. Such factors, according to the findings of this study, include supervisory relationship between supervisor and student, clinical learning environment, pedagogical atmosphere of the ward, leadership style of ward manager, and premises of nursing in the ward. Therefore, if the quality of these factors is maintained and improved upon, it could lead to a very high level of satisfaction with the clinical rotation of undergraduate nursing and midwifery students and hence improving nursing and midwifery clinical education in Ghana.

The findings of the study disprove the researchers initial thinking that student nurses and midwives have low levels of satisfaction with their clinical rotation experience and the clinical learning environment.

In summary, while all components of the clinical learning environment are important in determining students' satisfactions with their clinical rotation, nurses and midwives at the clinical sites should pay more attention to the premises of nursing in the ward. This has to do with the culture and values of nursing in the ward, information flow related to patients care, documentation of nursing care plans, recording of nursing procedures, and sufficient meaningful learning situations on the ward as recommended by Skaalvik et al. [14]. This is important because this study identified premises of nursing in the ward to be the most influential dimension of the clinical learning environment, which influences satisfaction with clinical rotation experience of undergraduate nursing and midwifery students. And it has been proven that satisfaction with clinical rotation experience enhances the students clinical skills acquisition, thereby making them better equipped when they graduate to offer quality nursing care to patients.

## 6. Limitations

The limitations of this study are that findings from this study could be peculiar to the study setting and may not be generalizable to other universities or jurisdictions. Moreover, due to the small number of the participants involved in the study, the study may have limited applicability. The study is also limited to third- and fourth-year nursing and midwifery students at one university. Therefore, the findings may not be generalized to all nursing and midwifery students in Ghana.

## Figures and Tables

**Table 1:** Demographic characteristics of participants.

Demographic characteristics	Frequency (*n* = 224)	Percentage (%)
*Ages*		
** **15–19	26	11.6
** **20–24	123	55.0
** **25–29	44	19.6
** **30–35	22	9.8
** **36–40	9	4.0

*Gender*		
** **Male	93	41.5
** **Female	131	58.5

*Marital Status*		
** **Single	176	78.6
** **Married	45	20.1
** **Devoice	3	1.3

*Religion*		
** **Christian	168	75
** **Moslem	50	22.3
** **Traditional	6	2.7

*Ethnicity*		
** **Mole Dagbani	88	39.3
** **Eve	17	7.6
** **Ga	21	9.4
** **Akan	62	27.7
** **Others	36	16.1

*Nursing Category*		
** **General nursing	160	71.4
** **Midwifery	64	28.6

*Financial Support*		
** **Self	67	29.9
** **Sponsorship	15	6.7
** **Parents	125	55.8
** **Guardian	17	7.6

*Level of Entry*		
** **Postdiploma	47	21
** **Generic	177	79

Source: Field Survey (2018).

**Table 2:** Respondents' level of satisfaction with clinical rotation experience.

Category	Frequency (224)	Percentage (100%)
Very low	1	0.4
Low	46	20.5
High	147	65.6
Very high	30	13.4

Source: Field Survey (2018).

**Table 3:** Association between selected demographic characteristics, clinical learning environment, and respondents' satisfaction with their clinical rotation experience.

Independent variables	*χ* ^2^	*Df*	*p-*value
*Demographic Characteristics*			
** **Age	15.254	12	0.241
** **Gender	5.346	3	0.118
** **Nursing students' category	4.482	3	0.201
** **Level of entry	5.002	3	0.116
*Clinical Learning Environment*			
** **Clinical learning environment	80.665	9	0.001^*∗*^

Dependent variable: clinical rotation experience, *χ*2: Fisher's exact test, *n* = 224, ^*∗*^significant at *p* ≤ 0.05.

**Table 4:** Respondents' level of satisfaction with the clinical learning environment.

Level of satisfaction	Frequency (224)	Percentage (100%)
Low	37	16.5
Very low	1	0.4
High	142	63.5
Very high	44	19.6

Source: Field Survey (2018).

**Table 5:** Strength of association between clinical learning environment and clinical rotation experience of respondents.

Independent variable	*R* ^2^	Unstandardized coefficients		Df	*p*-Value	95% C.I.	
		B	Standard error			Lower	Upper
Clinical learning environment	0.422	0.511	0.055	1.0	0.001^*∗*^	0.404	0.619
Constant		1.374	0.169			1.042	1.707

Dependent variable: clinical rotation experience, *n* = 224, ^*∗*^significant at *p* < 0.05.

**Table 6:** Relationship between students' satisfaction with clinical rotation experience and dimensions of the clinical learning environment.

Clinical learning environment dimensions	Spearman's rho	*p*-value
Pedagogical atmosphere of the ward	0.379	0.000^*∗*^
Leadership style of ward manager	0.340	0.000^*∗*^
Premises of nursing in the ward environment	0.501	0.000^*∗*^

Dependent variable: clinical Rotation Experience, ^*∗*^significant at *p* ≤ 0.05.

## Data Availability

The data used to support the findings of this study have not been made available because of a confidentiality clause in our informed consent that participants signed prior to the study. Participants were made to understand that data from them will only be used for the study and publication and will not be available to a third party.

## References

[1] Sebaee H. A. A., Al-Abdel Aziz E. M. A., Aziz N. T. (2017). Relationship between nursing students’ clinical placement satisfaction, academic self-efficacy and achievement. *IOSR Journal of Nursing and Health Science*.

[2] Esmaeili M., Cheraghi M. A., Salsali M., Ghiyasvandian S. (2014). Nursing students’ expectations regarding effective clinical education: a qualitative study. *International Journal of Nursing Practice*.

[3] Phillips K. F., Mathew L., Aktan N., Catano B. (2017). Clinical education and student satisfaction: an integrative literature review. *International Journal of Nursing Sciences*.

[4] Papastavrou E., Dimitriadou M., Tsangari H., Andreou C. (2016). Nursing students’ satisfaction of the clinical learning environment: a research study. *BMC Nursing*.

[5] Al-kandari F., Vidal V. L., Thomas D. (2009). Assessing clinical learning outcomes : a descriptive study of nursing students in Kuwait. *Nursing and Health Sciences*.

[6] Rajeswaran L. (2016). Clinical experiences of nursing students at a selected institute of health sciences in Botswana study design. *Health Science Journal*.

[7] Henderson A., Creedy D., Boorman R., Cooke M., Walker R. (2010). Development and psychometric testing of the clinical learning organisational culture survey (CLOCS). *Nurse Education Today*.

[8] Chan D. S. K. (2002). Associations between student learning outcomes from their clinical placement and their perceptions of the social climate of the clinical learning environment. *International Journal of Nursing Studies*.

[9] Sharif F., Masoumi S. (2005). A qualitative study of nursing student experiences of clinical practice. *BMC Nursing*.

[10] Saarikoski M., Leino-Kilpi H. (2002). The clinical learning environment and supervision by staff nurses: developing the instrument. *International Journal of Nursing Studies*.

[11] Chan D. (2001). Development of an innovative tool to assess hospital learning environments. *Nurse Education Today*.

[12] Abouelfettoh A., Mumtin S. (2015). Nursing students’ satisfaction with their clinical placement. *Journal of Scientific Research and Reports*.

[13] Bondas T. (2006). Paths to nursing leadership. *Journal of Nursing Management*.

[14] Skaalvik M. W., Normann H. K., Henriksen N. (2011). Clinical learning environment and supervision: experiences of Norwegian nursing students - a questionnaire survey. *Journal of Clinical Nursing*.

[15] Mantzoukas S., Jasper M. A. (2004). Reflective practice and daily ward reality: a covert power game. *Journal of Clinical Nursing*.

[16] Midgley K. (2006). Pre-registration student nurses perception of the hospital-learning environment during clinical placements. *Nurse Education Today*.

[17] Croxon L., Maginnis C. (2009). Evaluation of clinical teaching models for nursing practice. *Nurse Education in Practice*.

[18] Papp I., Markkanen M., von Bonsdorff M. (2003). Clinical environment as a learning environment: student nurses’ perceptions concerning clinical learning experiences. *Nurse Education Today*.

[19] Myrick F. (2015). Preceptorship and critical thinking in nursing education. *Journal of Nursing Education*.

[20] Goff A. M. (2009). *Stressors, Academic Performance, and Learned Resourcefulness in Baccalaureate Nursing Students*.

[21] James A., Chapman Y. (2009). Preceptors and patients – the power of two : nursing student experiences on their first acute clinical placement. *Contemporary Nurse*.

[22] Brown L., Herd K., Humphries G., Paton M. (2005). The role of the lecturer in practice placements: what do students think?. *Nurse Education in Practice*.

[23] Mikkonen I. (2005). *Clinical Learning as Experienced by Nursing Students*.

[24] Jamshidi N., Molazem Z., Sharif F., Torabizadeh C., Najafi Kalyani M. (2016). The challenges of nursing students in the clinical learning environment: a qualitative study. *The Scientific World Journal*.

[25] Mabuda B. T., Potgieter E., Alberts U. U. (2008). Student nurses’ experiences during clinical practice in the Limpopo Province. *Curationis*.

[26] Young K. H., Lee E., Sook E. (2012). *Effects of Simulation-Based Education on Communication Skill and Clinical Competence in Maternity Nursing Practicum*.

[27] Antohe I., Riklikiene O., Tichelaar E., Saarikoski M. (2016). Clinical education and training of student nurses in four moderately new European Union countries: assessment of students’ satisfaction with the learning environment. *Nurse Education in Practice*.

[28] Henderson A., Twentyman M., Eaton E., Creedy D., Stapleton P., Lloyd B. (2010). Creating supportive clinical learning environments: an intervention study. *Journal of Clinical Nursing*.

[29] Chuan O. L., Barnett T. (2012). Student, tutor and staff nurse perceptions of the clinical learning environment. *Nurse Education in Practice*.

[30] Papastavrou E., Lambrinou E., Tsangari H., Saarikoski M., Leino-Kilpi H. (2010). Student nurses experience of learning in the clinical environment. *Nurse Education in Practice*.

[31] Walker S., Rossi D., Anastasi J., Gray-Ganter G., Tennent R. (2016). Indicators of undergraduate nursing students’ satisfaction with their learning journey: an integrative review. *Nurse Education Today*.

[32] Atakro C. A., Gross J. (2016). Preceptorship versus clinical teaching partnership: literature review and recommendations for implementation in Ghana. *Advances in Nursing*.

[33] Atakro C. (2017). Experiences of student nurses and midwives at selected hospitals in the volta region of Ghana. *Journal of Education, Society and Behavioural Science*.

[34] Bhattacherjee A., American Studies Commons P. H. C., Commons E. (2012). *Social Science Research: Principles, Methods, and Practices*.

[35] Yamane T. (1967). *Statistics: An Introductory Analysis*.

[36] Johansson U. B., Kaila P., Ahlner-Elmqvist M., Leksell J., Isoaho H., Saarikoski M. (2010). Clinical learning environment, supervision and nurse teacher evaluation scale: psy- chometric evaluation of the Swedish version. *Journal of Advanced Nursing*.

[37] Macneen C. L., McCabe S. (2006). *Understanding Nursing Research*.

[38] Alspach G. (2006). Extending the synergy model to preceptorship. *Critical Care Nurse*.

[39] Zilembo M., Monterosso L. (2008). Towards a conceptual framework for preceptorship in the clinical education of undergraduate nursing students. *Contemporary Nurse*.

[40] Perli S., Brugnolli A. (2009). Italian nursing students’ perception of their clinical learning environment as measured with the CLEI tool. *Nurse Education Today*.

[41] Nepal B., Taketomi K., Ito Y. M. (2016). Nepalese undergraduate nursing students’ perceptions of the clinical learning environment, supervision and nurse teachers: a questionnaire survey. *Nurse Education Today*.

[42] Saarikoski M., Isoaho H., Warne T., Leino-Kilpi H. (2008). The nurse teacher in clinical practice: developing the new sub-dimension to the clinical learning environment and supervision (CLES) scale. *International Journal of Nursing Studies*.

[43] Hakim A. (2014). Nursing students’ satisfaction about their field of study. *Journal of Advances in Medical Education and Professionalism*.

[44] Hakim A. (2013). Nursing students ’ satisfaction. *International Journal of Nursing*.

[45] D’Souza M. S., Karkada S. N., Parahoo K., Venkatesaperumal R. (2015). Perception and satisfaction with the clinical learning environment among nursing students. *Nurse Education Today*.

[46] Akta Y. Y., Karabulut N. (2015). Nurse Education Today A Survey on Turkish nursing students’ perception of clinical learning environment and its association with academic motivation and clinical decision making. *Nurse Education Today Journal Science Direct*.

